# The clinical characterisation of the patient with primary psychosis aimed at personalisation of management

**DOI:** 10.1192/j.eurpsy.2021.34

**Published:** 2021-08-13

**Authors:** M. Maj

**Affiliations:** Department of Psychiatry, University of Campania L. Vanvitelli, Naples, Italy

## Abstract

The view that the management of the patient with primary psychosis should be personalized is currently endorsed by the vast majority of clinicians, but this personalization is lacking or inadequate in most clinical contexts. We describe systematically the salient domains that should be considered in the characterization of the individual patient with primary psychosis aimed at personalization of management. These include positive and negative symptom dimensions, other psychopathological components, onset and course, neurocognition and social cognition, neurodevelopmental indicators; social functioning, quality of life and unmet needs; clinical staging, antecedent and concomitant psychiatric conditions, physical comorbidities, family history, history of obstetric complications, early and recent environmental exposures, protective factors and resilience, and internalized stigma. For each domain, simple assessment instruments are identified that could be considered for use in clinical practice and included in standardized decision tools. A management of primary psychosis is encouraged which takes into account all the available treatment modalities whose efficacy is supported by research evidence, selects and modulates them in the individual patient on the basis of the clinical characterization, addresses the patient’s needs in terms of employment, housing, self-care, social relationships and education, and offers a focus on identity, meaning and resilience.

**Disclosure:**

No significant relationships.

